# Supplementary material for: Biophysical Characterization of the Binding Mechanism between the MATH Domain of SPOP and Its Physiological Partners
Source: Int J Mol Sci. 2023 Jun 14;24(12):10138. doi: 10.3390/ijms241210138 (PMC10298926; doi:10.3390/ijms241210138)
Supplement: Supplementary file 1 [file ijms-24-10138-s001.zip › ijms-2437231-SI.pdf]

Table S1. Binding kinetics parameters between SPOP MATH WT and the dansylated peptides Puc, MacroH2A and PTEN, at different ionic strengths.

[NaCl] mM	Puc			MacroH2A			PTEN		
	k_{on} ($\mu M^{-1} s^{-1}$)	k_{off} (s^{-1})	K_D (μM)	k_{on} ($\mu M^{-1} s^{-1}$)	k_{off} (s^{-1})	K_D (μM)	k_{on} ($\mu M^{-1} s^{-1}$)	k_{off} (s^{-1})	K_D (μM)
**0**	19 ±1	26.1 ± 0.1	1.4 ± 0.4	3 ± 2	21 ± 2	7.8 ± 0.4	5.5 ± 0.5	113 ±1	20.8 ± 0.4
**10**	-	-	-				2.3 ± 0.2	200 ± 4	88 ± 9
**25**	-	-	-				0.8 ± 0.1	234 ± 2	290 ± 10
**50**	3.91 ± 0.09	23.0 ± 0.5	5.9 ± 0.1	0.95 ± 0.09	20.4 ± 0.5	21.6 ± 0.5	0.5 ± 0.5	214 ± 1	450 ± 10
**100**	2.5 ± 0.1	22.7 ± 0.5	9.1 ± 0.1	0.8 ± 0.1	18.2 ± 0.5	22.3 ± 0.5	-	-	-
**200**	1.46 ± 0.08	22.0 ± 0.5	15.0 ± 0.5	0.98 ± 0.08	14.4 ± 0.5	14.6 ± 0.5	-	-	-
**500**	1.9 ± 0.2	23 ± 1	12.0 ± 0.5	0.5 ± 0.2	15 ± 1	30.7 ± 0.5	-	-	-

The calculated parameters were obtained using pseudo-first order analysis, as described in the methods Section.

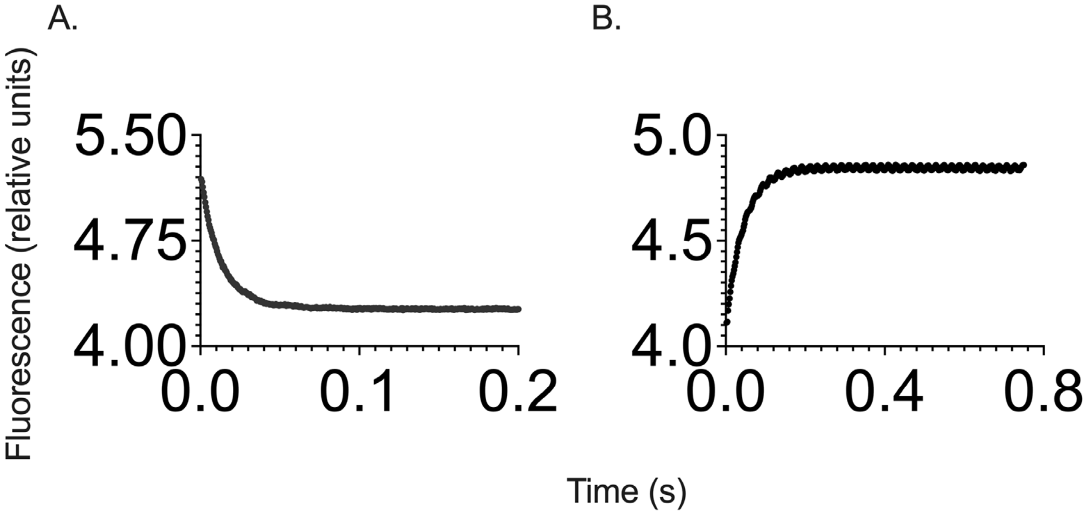

Figure S1. A typical binding (panel A) and displacement (panel B) fluorescence time course observed for the MATH domain when recognizing its substrates. The reported time courses refer to the average of 3-6 independent measurements.
